# Supplementary material for: Treatment of Acute Respiratory Distress Syndrome Caused by COVID-19 with Human Umbilical Cord Mesenchymal Stem Cells
Source: Int J Mol Sci. 2023 Feb 23;24(5):4435. doi: 10.3390/ijms24054435 (PMC10003440; doi:10.3390/ijms24054435)
Supplement: Supplementary file 1 [file ijms-24-04435-s001.zip › ijms-2104484-supplementary/Supplementary_Figure_S1-S6.pdf]

Supplementary Material

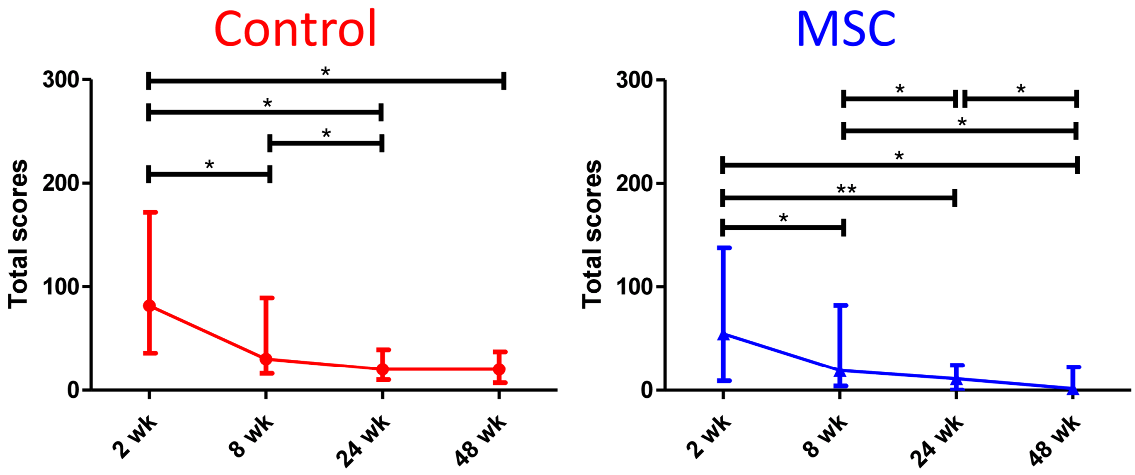

Supplementary Figure S1. Dynamic changes in lung CT scores in patients with COVID-19. Data are presented as a median and IQR. Paired t-test was used for comparing related values of time-depending events: *, p≤0.05; **, p≤0.01. The red line represents the Control group; the blue line represents the MSC group.

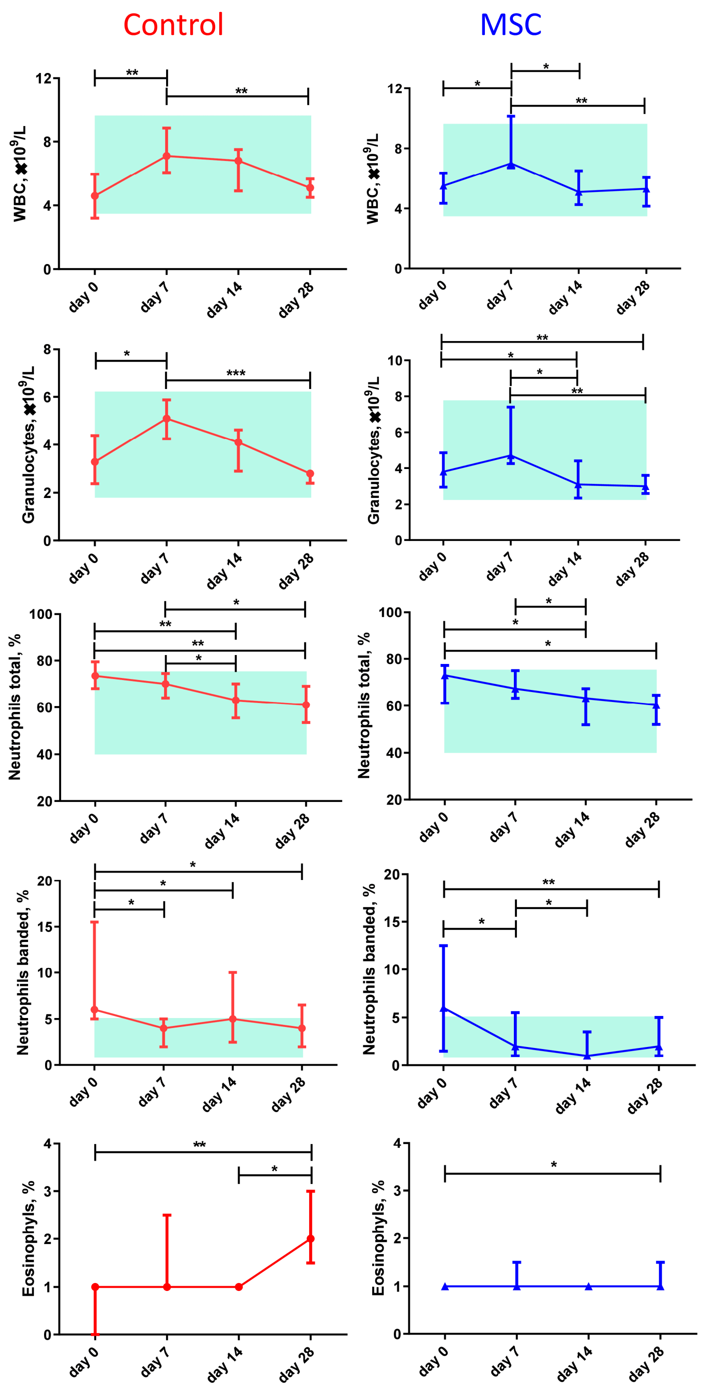

Supplementary Figure S2. Dynamic changes in in hematological parameters in patients with COVID-19. A light green rectangle denotes the normal range. Data are presented as the median and interquartile range. Wilcoxon signed-rank test: *, p≤0.05; **, p≤0.01; ***, p≤0.001. WBC, white blood cells; ESR, erythrocyte sedimentation rate. The red line represents the Control group; the blue line represents the MSC group.

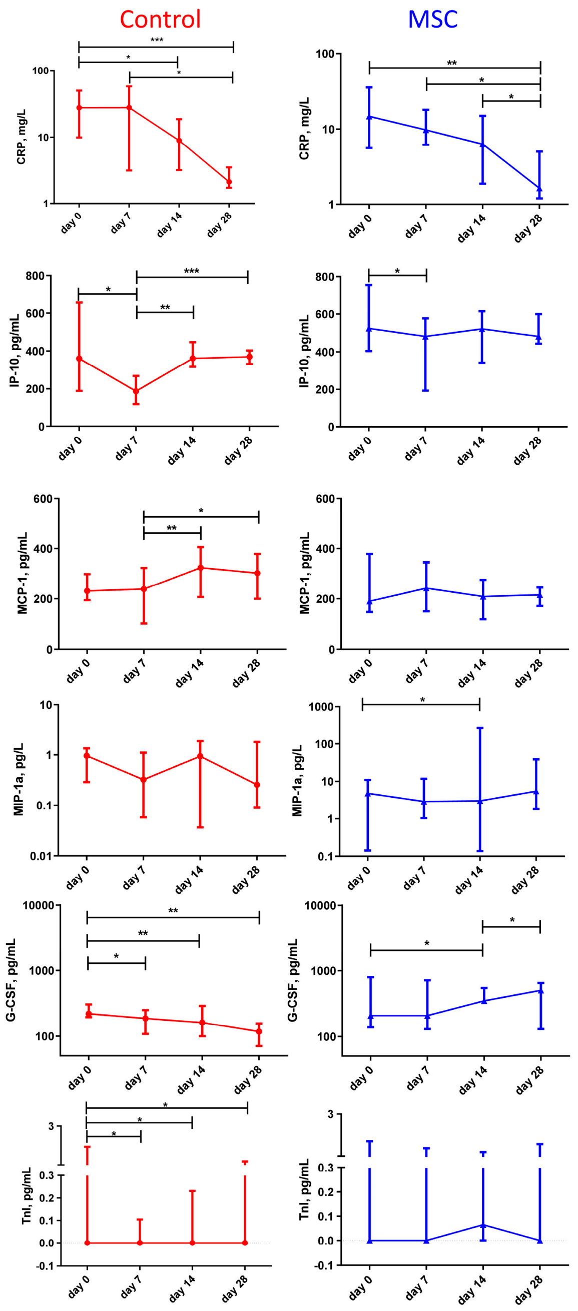

Supplementary Figure S3. Dynamic changes in cytokine levels in patients with COVID-19. Data are presented as a median and interquartile range. Wilcoxon signed-rank test for comparing time-dependent events: *, p≤0.05; **, p≤0.01; ***, p≤0.001. The red line represents the Control group; the blue line represents the MSC group. CRP, C-reactive protein; IP-10, Interferon gamma-induced protein 10; MCP-1, Monocyte chemoattractant protein-1; MIP-1α, Macrophage Inflammatory Protein-1 Alpha; G-CSF, Granulocyte colony-stimulating factor; IL-10, interleukin 10; IL-2, interleukin 2; IL-6, interleukin 6; NT-proBNP, N-terminal prohormone of brain natriuretic peptide; TnI, troponin I; SP-D, surfactant D; RAGE, receptor for advanced glycation endproducts.

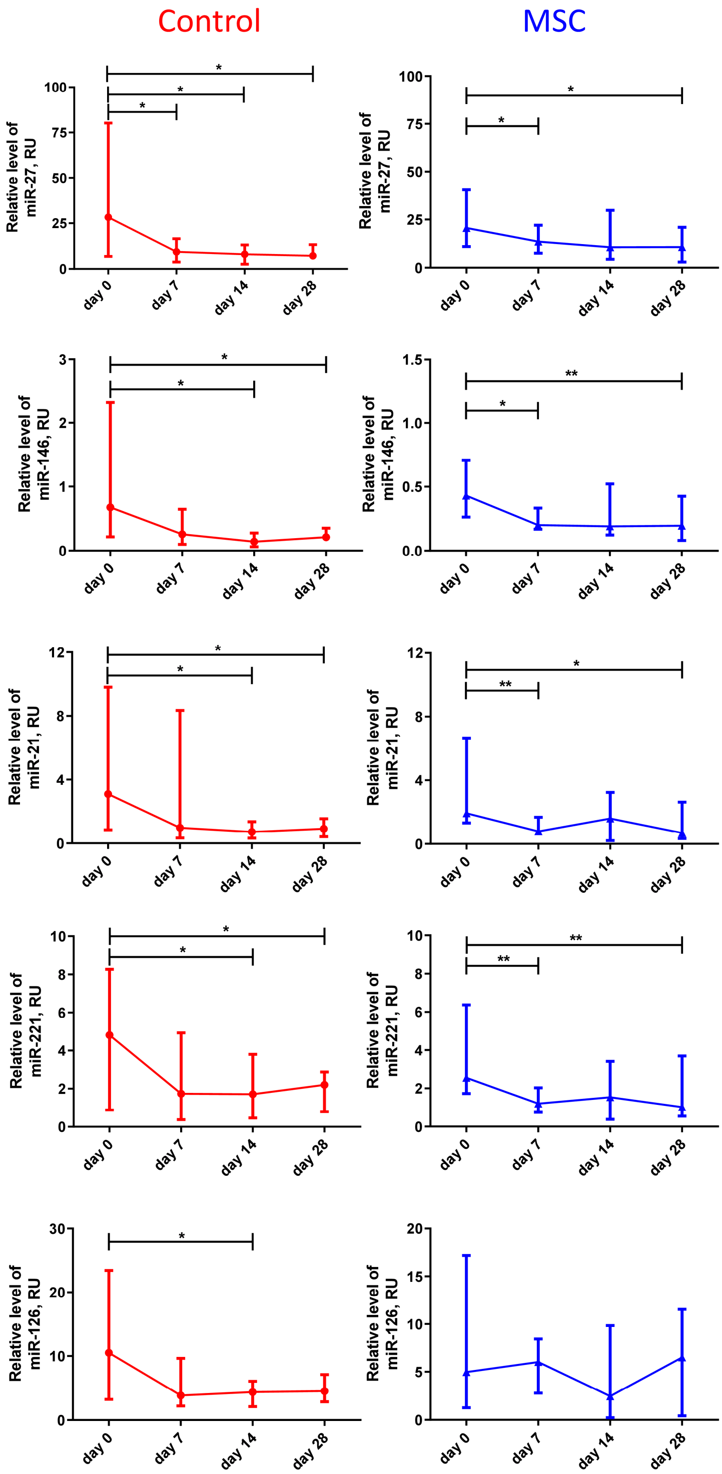

Supplementary Figure S4. Dynamic changes in miRNAs expression level in patients with COVID-19. Data are presented as a median and IQR. Wilcoxon signed-rank test for comparing time-dependent events: *, p≤0.05; **, p≤0.01. The red line represents the Control group; the blue line represents the MSC group.

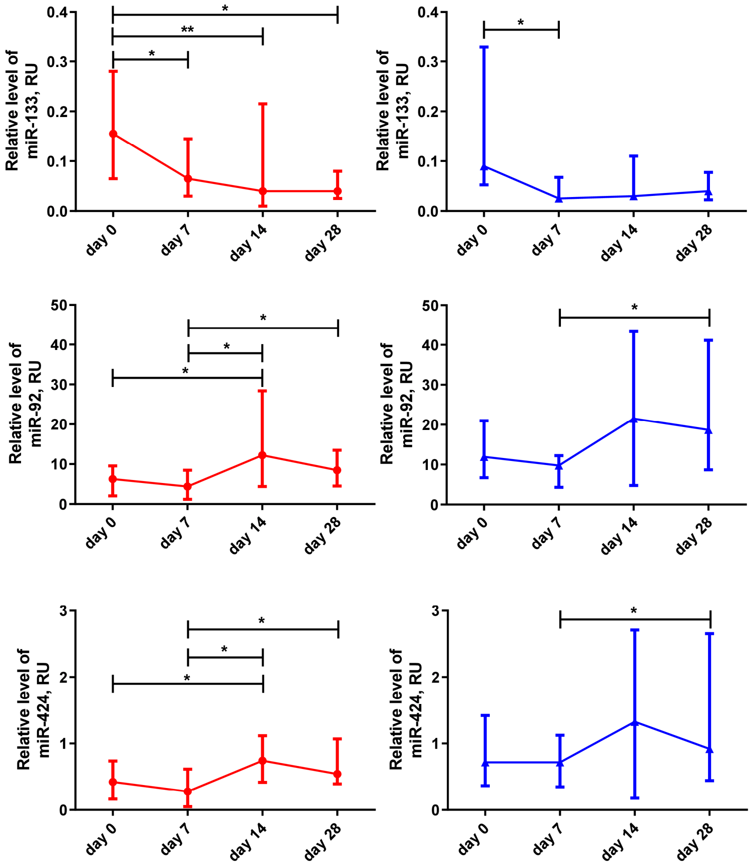

Supplementary Figure S4 (continued). Dynamic changes in miRNAs expression level in patients with COVID-19. Data are presented as a median and IQR. Wilcoxon signed-rank test for comparing time-dependent events: *, $p \leq 0.05$; **, $p \leq 0.01$. The red line represents the Control group; the blue line represents the MSC group.

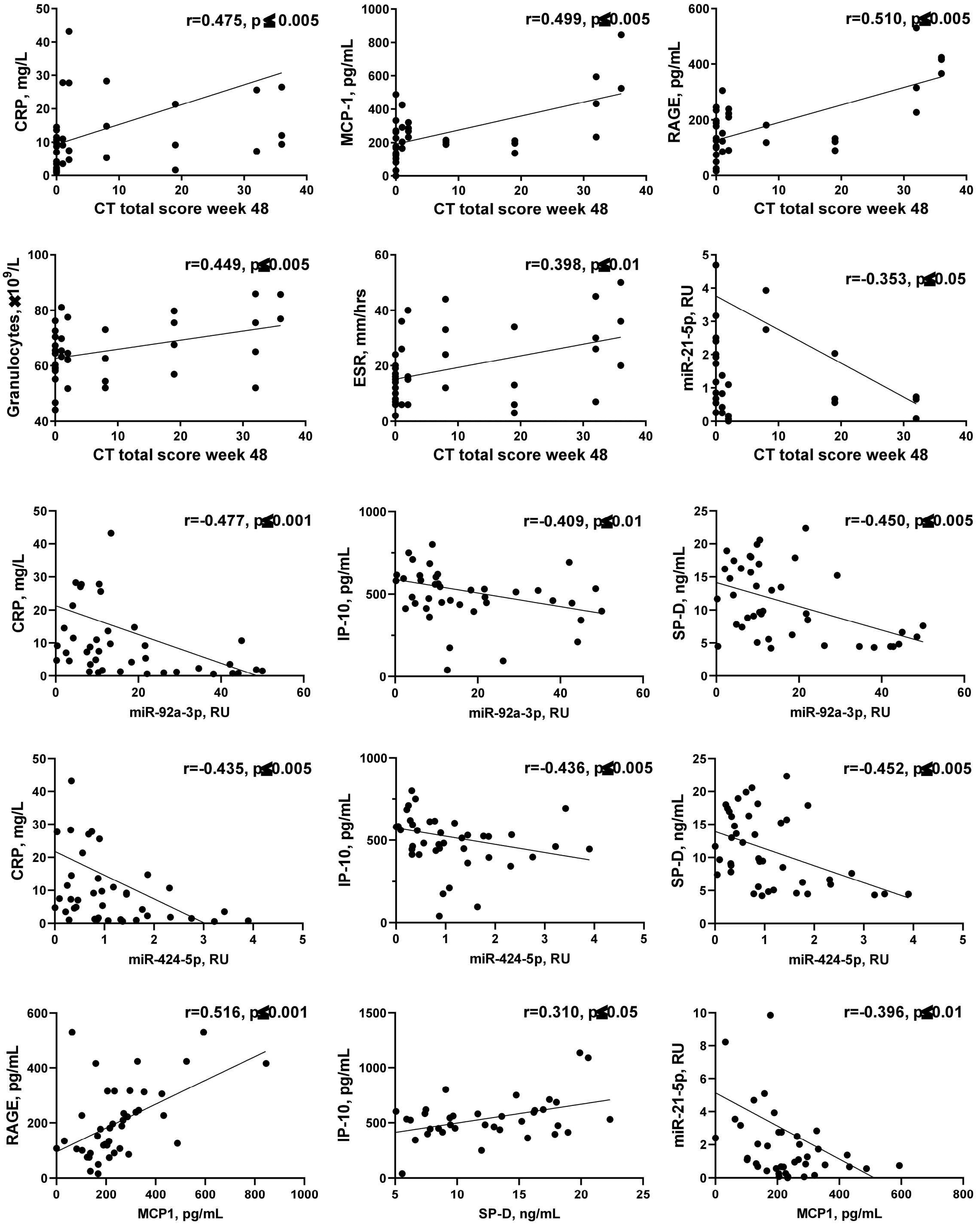

Supplementary Figure S5. Some correlation between lung CT total scores, blood parameters, cytokines, and miRNA in COVID-19 patients. All data on the correlation analysis are available in **Table S1.**

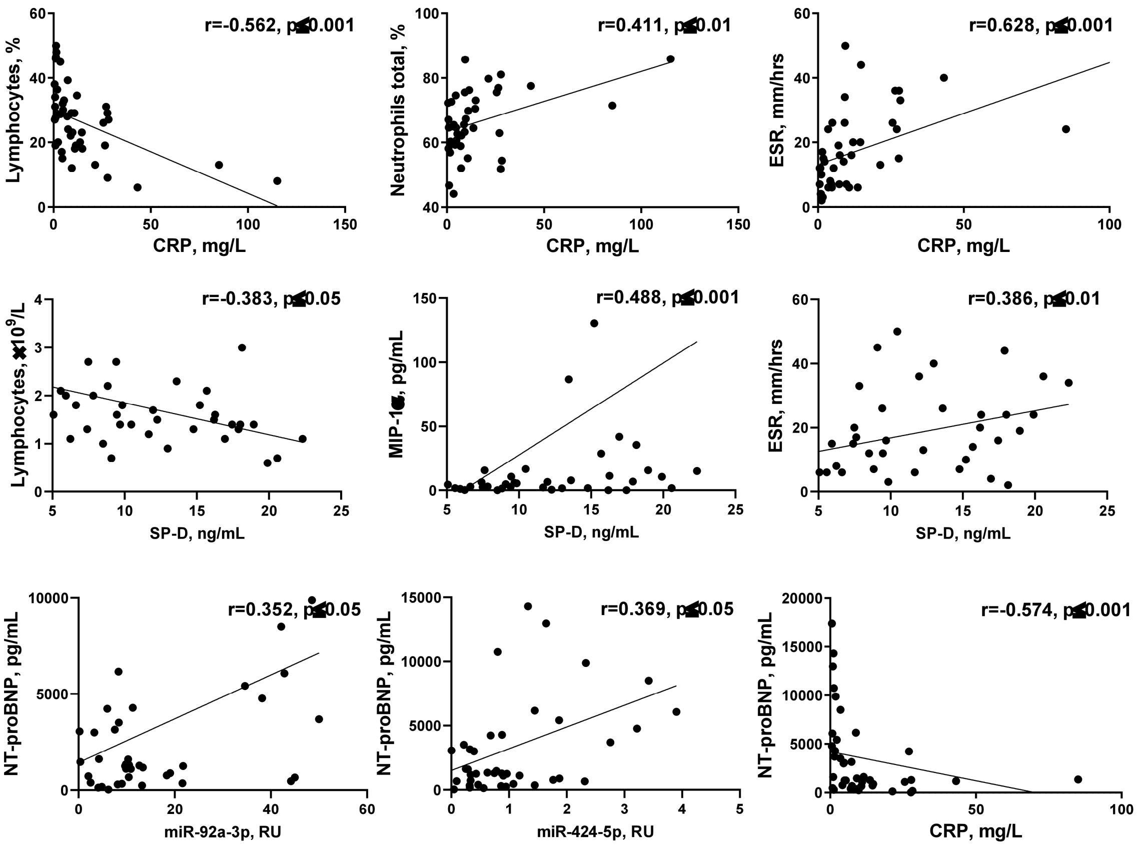

Supplementary Figure S5 (continued). Some correlation between lymphocyte subsets, cytokines, and miRNA in COVID-19 patients. All data on the correlation analysis are available in **Table S1.**

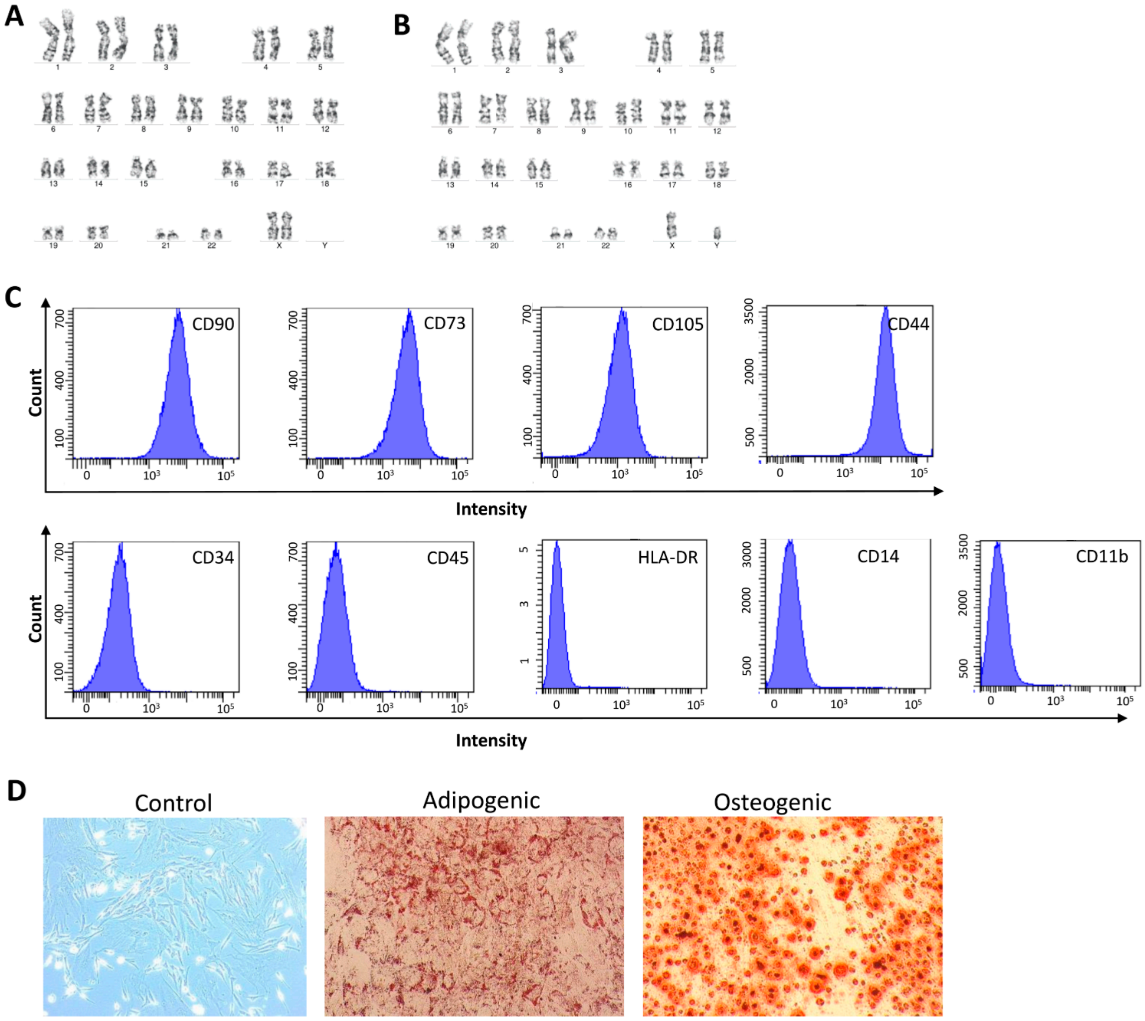

Supplementary Figure S6. Characteristics of UC-MSCs. A representative example of normal female (**A**) and male (**B**) karyotype of a UC-MSCs line at P. (**C**)**,** UC-MSCs were positive for CD90, CD73, CD105 and negative for CD34 and CD45 at P3. (**D**)**,** Abundance of lipid vacuoles and single nodules of extracellular calcium matrix were detected in UC-MSCs cultures at P3 after adipogenic and osteogenic induction; Oil Red O and Alizarin Red S staining, magnification ×50.
