# Supplementary material for: Treatment of Acute Respiratory Distress Syndrome Caused by COVID-19 with Human Umbilical Cord Mesenchymal Stem Cells
Source: Int J Mol Sci. 2023 Feb 23;24(5):4435. doi: 10.3390/ijms24054435 (PMC10003440; doi:10.3390/ijms24054435)
Supplement: Supplementary file 1 [file ijms-24-04435-s001.zip › ijms-2104484-supplementary/Supplementary_Table_S2.pdf]

Supplementary Material

Supplementary Table S2: Primer sequences used for miRNAs detection.

Name	Sequence for	Sequence rev
hsa-miR-21-5p	TAGCTTATCAGACTGATGTTGA	Universal reverse primer (kit provided)
hsa-miR-27a-3p	TTCACAGTGGCTAAGTTCCGC	
hsa-miR-126-3p	TCGTACCGTGAGTAATAATGCG	
hsa-miR-133a-3p	TTTGGTCCCCTTCAACCAGCTG	
hsa-miR-146a-5p	TGAGAACTGAATTCCATGGGTT	
hsa-miR-221-3p	AGCTACATTGTCTGCTGGGTTTC	
hsa-miR-92a-3p	TATTGCACTTGTCCCGGCCTGT	
has-miR-124-3p	TAAGGCACGCGGTGAATGCCAA	
hsa-miR-424a-5p	CAGCAGCAATTCATGTTTTGAA	
U6	CTCGCTTCGGCAGCACA	AACGCTTCACGAATTTGCGT
